# Activity of five antimicrobial peptides against periodontal as well as non-periodontal pathogenic strains

**DOI:** 10.1080/20002297.2020.1829405

**Published:** 2020-10-07

**Authors:** Katharina Enigk, Holger Jentsch, Arne C. Rodloff, Klaus Eschrich, Catalina-Suzana Stingu

**Affiliations:** a Institute for Medical Microbiology and Epidemiology of Infectious Diseases, University Hospital of Leipzig, Leipzig, Germany; b Center for Periodontology, Department of Cariology, Endodontology and Periodontology, University of Leipzig, Leipzig, Germany; c Institute of Biochemistry, Medical Faculty, University of Leipzig, Leipzig, Germany

**Keywords:** Antimicrobial peptide, antimicrobial alternative, periodontal bacteria, oral bacteria, agar dilution method

## Abstract

**Background**: Due to the increasing emergence of multi-resistant bacteria the search for alternative antimicrobial substances is of high interest. Promising agents are antimicrobial peptides which are host defense molecules of the innate immune system in a wide range of different species.

**Objectives**: The aim of this study was to assess the activity of nisin, melittin, lactoferrin, parasin-1 and LL-37 against 35 oral bacteria and *Candida albicans* employing the gold standard method for anaerobic susceptibility testing.

**Methods**: The activity of the peptides was determined by an agar dilution method under anaerobic and aerobic conditions. The test media contained final peptide concentrations between 0.125 µg/ml and 8 µg/ml (melittin, lactoferrin, parasin-1, LL-37) and between 0.125 µg/ml and 128 µg/ml (nisin).

**Results**: Nisin completely inhibited the growth of *Megasphaera* sp., *Bifidobacterium longum, Parvimonas micra, Actinomyces israelii, Actinomyces naeslundii, Actinomyces odontolyticus, Prevotella intermedia, Streptococcus anginosus, Streptococcus constellatus* and *Staphylococcus aureus*. Melittin and lactoferrin reduced the growth of *Megasphaera* sp., *P. micra, B. longum* (melittin) and *Selenomonas flueggei* (lactoferrin). Parasin-1 and LL-37 showed no activity.

**Conclusion**: AMPs, especially nisin and to a smaller degree lactoferrin, might be promising alternatives to antibiotics because of their antimicrobial activity, high resistance to environmental conditions and partially low costs.

## Introduction

The key factor in the etiology of periodontitis is the microbial biofilm formed on the tooth surface. The mechanical destruction of dental calculus and biofilm in form of scaling and root planing is the gold standard of the clinical periodontal treatment [1,2]. Additionally, antiseptics such as chlorhexidine or antibiotics are used as chemical adjuncts [3,4]. Systemic antibiotics offer a significant clinical benefit only for certain groups of patients and should be restrictively used in order to prevent the emergence of resistant bacteria [5]. In 2017, the World Health Organization (WHO) declared drug-resistant bacteria as the greatest threat to human health [6]. As a result, the search for alternative antimicrobial substances with a lower risk of developing bacterial resistance is of high interest.

Lately, a group of heterogenic antimicrobial peptides (AMPs) came into the focus of research. These host defense molecules are part of the innate immune system in a wide range of different species, including vertebrates, invertebrates, bacteria, plants or fungi [7,8]. According to an American online database over 3,000 AMPs are isolated and characterized [9]. Most of them are produced by animals (around 75%), are cationic and have a peptide length between 20 and 50 amino acids [9]. In addition to their antimicrobial activity some peptides also have other functions i.e. in wound healing, immune modulation or apoptosis [10–12]; some others exhibit anticancer activity [13].

For this study we have chosen five AMPs of different origin: 1. Nisin is produced by the Gram-positive species *Lactococcus lactis* [14]. 2. Melittin is the main component of honey bee venom [15]. 3. Parasin-1 is produced by skin cells of the catfish, *Parasilurus asotus* [16]. 4. Lactoferrin is present in mucosal secretions, like milk, saliva or lacrimal fluid and is produced by epithelial cells of glands [17]. 5. Cathelicidin, hCAP-18, is produced by human leukocytes as well as epithelial cells and is processed extracellularly by a serine protease to form the active LL-37 [18]. Common features of these AMPs are their selective antimicrobial activity and at the same time a limited propensity to induce resistance. This makes them promising agents in therapy research.

The antimicrobial mechanism of the peptides involves an initial non-specific interaction with components of the bacterial membrane like negatively charged phospholipids, lipopolysaccharide or teichoic acids [19]. The insertion of the peptides into the lipid bilayer compromises the barrier effect of the bacterial membrane leading to a loss of transmembrane potential, to a leakage of cytoplasmic components and finally to cell death [20–22].

Previous studies have already determined the antimicrobial activity of peptides against some bacterial species. The aim of this study was to assess the activity of five AMPs against periodontal pathogens and other bacteria that can occur in the oral cavity employing the gold standard method for anaerobic susceptibility testing.

## Materials and methods

### Bacterial strains and preparation of inoculum

Thirty-six different microbial strains (23 reference strains from the American Type Culture Collection (ATCC) or the German Collection of Microorganism and Cell Cultures (DSMZ) as well as 13 clinical strains) were used to test the five AMPs (Table 1). All bacterial strains were grown on Brucella agar with 5 mg of hemin/liter, 1 mg of vitamin K/liter and 5% sheep blood and *Candida albicans* was grown on Sabouraud agar. Anaerobic species were cultivated at 37°C in an anaerobic chamber (Whitley MG 1000, anaerobic workstation, Meintrup DWS Laborgeräte GmbH, Germany) containing 80% N_2_, 15% CO_2_ and 5% H_2_. All facultative anaerobes were cultivated in presence of 5% CO_2_ (Heracell^TM^ 150i, CO_2_ incubator, Thermo Scientific^TM^, USA).

**Table 1.** Microbial strains used in this study for testing the activity of 5 antimicrobial peptides.

Species	Reference
*Actinomyces israelii*	ATCC 12102
*Actinomyces johnsonii*	clinical strain
*Actinomyces naeslundii*	ATCC 12104
*Actinomyces odontolyticus*	DSM 43331
*Actinomyces oris*	DSM 23056
*Aggregatibacter actinomycetemcomitans*	ATCC 29523
*Atopobium parvulum*	clinical strain
*Bifidobacterium longum*	clinical strain
*Campylobacter concisus*	clinical strain
*Campylobacter rectus*	DSM 3260
*Dialister pneumosintes*	DSM 11619
*Eikenella corrodens*	ATCC 23834
*Enterobacter cloacae*	clinical strain
*Enterococcus faecalis*	ATCC 29212
*Escherichia coli*	ATCC 25922
*Fusobacterium nucleatum* ssp. *fusiforme*	ATCC 51190
*Fusobacterium nucleatum* ssp. *nucleatum*	ATCC 25586
*Fusobacterium nucleatum* ssp. *polymorphum*	ATCC 10953
*Fusobacterium nucleatum* ssp. *vincentii*	ATCC 49256
*Megasphaera* sp.	DSM 102144
*Parvimonas micra*	clinical strain
*Porphyromonas gingivalis*	clinical strain
*Prevotella intermedia*	DSM 20706
*Prevotella loescheii*	clinical strain
*Prevotella melaninogenica*	clinical strain
*Prevotella nigrescens*	DSM 13386
*Pseudomonas aeruginosa*	ATCC 27853
*Selenomonas flueggei*	clinical strain
*Selenomonas sputigena*	clinical strain
*Slackia exigua*	clinical strain
*Staphylococcus aureus*	ATCC 29213
*Streptococcus anginosus*	DSM 20563
*Streptococcus constellatus* ssp. *constellatus*	DSM 20575
*Streptococcus mutans*	DSM 20523
*Veillonella parvula*	clinical strain
*Candida albicans*	DSM 11949

The inoculum of each tested strain was prepared fresh on the day of test. The final inoculum density was adjusted to approximately 3 × 10^5^ CFU for anaerobic species and 1.5 × 10^5^ CFU for facultative anaerobes as well as for *C. albicans*. The identification of all reference and clinical strains was confirmed using the VITEK-MS system (bioMérieux, France) or the sequence analysis of the 16S rRNA gene.

### Antimicrobial peptides and preparation of media

Antimicrobial peptides used in this study were nisin, melittin, human lactoferrin (Sigma-Aldrich Chemie GmbH, Germany), LL-37 and parasin-1 (Isca Biochemicals, UK). Stock solutions were prepared on the day of testing. Nisin, melittin and lactoferrin were solved in distilled water. LL-37 and parasin-1 were dissolved in PBS and diluted in distilled water according to the manufacturer’s recommendations.

The antimicrobial activity of the peptides was determined by an agar dilution method according to the European Committee for Antimicrobial Susceptibility Testing (EUCAST) using Brucella agar supplemented with 5 mg of hemin/liter, 1 mg of vitamin K/liter and 5% sheep blood (pH 7) [23]. Test media were prepared by adding geometric dilutions of the peptides to containing final concentrations of the antimicrobial agent between 0.125 µg/ml and 8 µg/ml (melittin, lactoferrin, LL-37, parasin-1) and between 0.125 µg/ml and 128 µg/ml (nisin). For each experiment three agar plates (two of them being incubated anaerobically and one aerobically) with the same concentration of peptides were inoculated.

### Agar dilution method

A semiautomated replicator device (A400 Multipoint inoculator, Bachofer GmbH, Germany) was used to inoculate the test media. At the beginning and end of each test, three agar plates with no peptide were inoculated as growth control, under aerobic and anaerobic conditions, respectively.

After 4 days of incubation we evaluated the growth on the peptide agar plates and compared it with the control plates. MIC was defined as the lowest concentration of the peptide that completely inhibited visible growth of microorganism. To differentiate between different levels of growth reduction observed growth categories were defined (Table 2). All tests described above were performed in duplicate using newly cultivated microorganisms.

**Table 2.** Growth categories of different levels of reduced growth of the bacteria on the agar peptide plates.

Growth category	Growth on agar plates
Complete inhibition	no colonies
Significant growth reduction	few or sparse countable colonies
Slight growth reduction	confluent growth with declining tendency
No growth reduction	confluent growth comparable to growth control

## Results

The growth control plates showed good growth of all microorganisms tested.

Nisin, melittin and lactoferrin showed an antimicrobial activity against some strains. Of all AMPs tested in this study, nisin showed the highest activity (Tables 3 and 4). It was the only peptide that completely inhibited growth at concentrations under 8 µg/ml. This applied to the growth of *Megasphaera* sp., *Bifidobacterium longum* and *Parvimonas micra*. Higher concentrations up to 128 µg/ml also completely inhibited the growth of *Actinomyces israelii, Actinomyces naeslundii, Actinomyces odontolyticus, Prevotella intermedia, Streptococcus anginosus, Streptococcus constellatus* ssp. *constellatus* as well as *Staphylococcus aureus*. The MIC-values measured on replicated tests did not differ more than one dilution.

**Table 3.** Bacterial growth on agar plates with different concentrations of the five AMPs cultivated in anaerobic atmosphere. Concentrations between 0.125 µg/ml and 8 µg/ml of all peptides were tested. In addition, nisin concentrations up to 128 µg/ml were tested.

Species	Peptide [µg/ml]	0,125	0,25	0,5	1	2	4	8	16	32	64	128
*Megasphaera* sp.	Nisin	+	+	+	±	-	-	-	-	-	-	-
	Lactof	+	+	+	+	+	+	+				
	Melit	+	+	+	+	+	+	+				
	Para-1	+	+	+	+	+	+	+				
	LL-37	+	+	+	+	+	+	+				
*P. micra*	Nisin	+	+	+	+	+	+	±	-	-	-	-
	Lactof	+	+	+	+	+	+	+				
	Melit	+	+	+	+	+	+	+				
	Para-1	+	+	+	+	+	+	+				
	LL-37	+	+	+	+	+	+	+				
*B. longum*	Nisin	+	+	+	±	-	-	-	-	-	-	-
	Lactof	+	+	+	+	+	+	+				
	Melit	+	+	+	+	+	+	+				
	Para-1	+	+	+	+	+	+	+				
	LL-37	+	+	+	+	+	+	+				
*A. israelii*	Nisin	+	+	+	+	+	+	+	-	-	-	-
	Lactof	+	+	+	+	+	+	+				
	Melit	+	+	+	+	+	+	+				
	Para-1	+	+	+	+	+	+	+				
	LL-37	+	+	+	+	+	+	+				
*A. naeslundii*	Nisin	+	+	+	+	+	+	+	+	-	-	-
	Lactof	+	+	+	+	+	+	+				
	Melit	+	+	+	+	+	+	+				
	Para-1	+	+	+	+	+	+	+				
	LL-37	+	+	+	+	+	+	+				
*A. odontolyticus*	Nisin	+	+	+	+	+	+	+	-	-	-	-
	Lactof	+	+	+	+	+	+	+				
	Melit	+	+	+	+	+	+	+				
	Para-1	+	+	+	+	+	+	+				
	LL-37	+	+	+	+	+	+	+				
*S. aureus*	Nisin	+	+	+	+	+	+	+	+	+	+	-
	Lactof	+	+	+	+	+	+	+				
	Melit	+	+	+	+	+	+	+				
	Para-1	+	+	+	+	+	+	+				
	LL-37	+	+	+	+	+	+	+				
*S. anginosus*	Nisin	+	+	+	+	+	+	+	+	+	+	-
	Lactof	+	+	+	+	+	+	+				
	Melit	+	+	+	+	+	+	+				
	Para-1	+	+	+	+	+	+	+				
	LL-37	+	+	+	+	+	+	+				
*S. constellatus* ssp. *con.*	Nisin	+	+	+	+	+	+	+	+	+	-	-
	Lactof	+	+	+	+	+	+	+				
	Melit	+	+	+	+	+	+	+				
	Para-1	+	+	+	+	+	+	+				
	LL-37	+	+	+	+	+	+	+				
*P. intermedia*	Nisin	+	+	+	+	+	+	+	+	+	-	-
	Lactof	+	+	+	+	+	+	+				
	Melit	+	+	+	+	+	+	+				
	Para-1	+	+	+	+	+	+	+				
	LL-37	+	+	+	+	+	+	+				
*A. johnsonii, A. oris, A. actinomycetemcomitans, A. parvulum, C. concisus, C. rectus, D. pneumosintes, E. corrodens, E. cloacae, E. faecalis, E. coli, F nucleatum* (*fusiforme, nucleatum, polymorphum, vincentii*), *P. gingivalis, P. loescheii, P. melaninogenica, P. nigrescens, S. flueggei, S. sputigena, S. exigua, S. mutans, V. parvula, C. albicans*	Nisin	+	+	+	+	+	+	+	+	+	+	+
	Lactof	+	+	+	+	+	+	+				
	Melit	+	+	+	+	+	+	+				
	Para-1	+	+	+	+	+	+	+				
	LL-37	+	+	+	+	+	+	+				

‘+’ – growth categories ii, iii, iv; ‘-’ – growth category i; ‘±’ – growth category i on at least one agar plate in at least one experiment; ‘*empty table element*’ – not measured; ‘Lactof’ – Lactoferrin; ‘Melit’ – Melittin; ‘Para-1’ – Parasin-1

*P. aeruginosa* was not cultivated anaerobically.

**Table 4.** Bacterial growth on agar plates with different concentrations of nisin cultivated under aerobic atmosphere. In test 1 and 2 nisin concentrations between 0.125 µg/ml and 8 µg/ml were tested. In test 3 and 4 nisin concentrations between 0.5 µg/ml and 128 µg/ml were tested. (Bacteria with no growth inhibition are not shown.).

Nisin [µg/ml] aerobic atmosphere		0.125	0.25	0.5	1	2	4	8	16	32	64	128
*A. israelii*	Test 1	+	+	+	+	+	+	+				
	Test 2	+	+	+	+	+	+	+				
	Test 3			+	+	+	+	+	+	-	-	-
	Test 4			+	+	+	+	+	+	-	-	-
*A. naeslundii*	Test 1	+	+	+	+	+	+	+				
	Test 2	+	+	+	+	+	+	+				
	Test 3			+	+	+	+	+	+	-	-	-
	Test 4			+	+	+	+	+	+	+	-	-
*A. odontolyticus*	Test 1	+	+	+	+	+	+	+				
	Test 2	+	+	+	+	+	+	+				
	Test 3			+	+	+	+	+	-	-	-	-
	Test 4			+	+	+	+	+	-	-	-	-
*S. aureus*	Test 1	+	+	+	+	+	+	+				
	Test 2	+	+	+	+	+	+	+				
	Test 3			+	+	+	+	+	+	+	-	-
	Test 4			+	+	+	+	+	+	+	-	-
*S. anginosus*	Test 1	+	+	+	+	+	+	+				
	Test 2	+	+	+	+	+	+	+				
	Test 3			+	+	+	+	+	+	+	-	-
	Test 4			+	+	+	+	+	+	+	+	-
*S. constellatus* ssp. *con.*	Test 1	+	+	+	+	+	+	+				
	Test 2	+	+	+	+	+	+	+				
	Test 3			+	+	+	+	+	+	+	-	-
	Test 4			+	+	+	+	+	+	+	-	-

‘+’ – growth categories ii, iii, iv; ‘-’ – growth category i; ‘*empty table element*’ – not measured.

Nisin at a concentration of 128 µg/ml also resulted in growth reduction of other bacteria without completely inhibiting their growth (Table 5). The growth of *Veillonella parvula, Atopobium parvulum, Slackia exigua, Prevotella nigrescens* and *Prevotella loescheii* was significantly reduced, while the growth of *Selenomonas flueggei* and *Porphyromonas gingivalis* was only slightly inhibited.

**Table 5.** Antimicrobial activity of nisin, melittin and lactoferrin tested by the agar dilution method. Reduced growth of bacteria on the agar peptide plates was compared with the growth control plates and differentiated into different levels (i, ii, iii) of reduced growth. (Bacteria with no growth reduction (iv) are not shown.).

Peptide	Strain	i) complete inhibition, MIC in [µg/ml]	ii) significant growth reduction, concentration in [µg/ml]	iii) slight growth reduction, concentration in [µg/ml]
Nisin	*A. israelii*	16, 32		
	*A. naeslundii*	32, 64		
	*A. odontolyticus*	16		
	*A. parvulum*		128	
	*B. longum*	1, 2		
	*Megasphaera* sp.	1, 2		
	*P. micra*	8, > 8		
	*P. gingivalis*			128
	*P. intermedia*	64		
	*P. loescheii*		128	
	*P. nigrescens*		128	
	*S. flueggei*			128
	*S. exigua*		128	
	*S. anginosus*	64, 128		
	*S. aureus*	64, 128		
	*S. constellatus* ssp. *con.*	64		
	*V. parvula*		128	
Melittin	*B. longum*		8	
	*Megasphaera* sp.		8	
	*P. micra*		8	
Lactoferrin	*Megasphaera* sp.		8	
	*P. micra*			8
	*S. flueggei*			8

No growth reduction or inhibition at 128 µg/ml of nisin showed *Actinomyces johnsonii, Actinomyces oris, Aggregatibacter actinomycetemcomitans, Campylobacter concisus, Campylobacter rectus, Dialister pneumosintes, Eikenella corrodens, Enterobacter cloacae, Enterococcus faecalis, Escherichia coli, Fusobacterium nucleatum* (ssp. *fusiforme*, ssp. *nucleatum*, ssp. *polymorphum*, ssp. *vincentii), Prevotella melaninogenica, Pseudomonas aeruginosa, Selenomonas sputigena, Streptococcus mutans* and *C. albicans*.

Melittin and lactoferrin did not completely inhibit growth of any bacteria (Table 3). But at a concentration of 8 µg/ml melittin significantly reduced the growth of *Megasphaera* sp., *B. longum* as well as *P. micra*. Likewise, lactoferrin also significantly reduced the growth of *Megasphaera* sp. but only slightly inhibited the growth of *P. micra* and *S. flueggei* (Table 5).

LL-37 and parasin-1 showed no activity against the bacteria tested (Table 3). Not even a slight growth reduction at concentrations of 8 µg/ml was observed.

## Discussion

In this study the activity of five AMPs of different origin was tested. The AMP concentrations were chosen based on previous studies and their possible usefulness for clinical purposes. Periodontopathogenic bacteria (mostly anaerobes) from different microbial complexes described by Socransky and Haffajee as well as non-periodontopathogenic strains were tested [24].

The antimicrobial activity of the peptides was determined by an agar dilution method according to EUCAST which represents the gold standard for susceptibility testing of anaerobic bacteria [23]. Although the agar dilution method is labor intensive and time-consuming, the advantage in comparison to the broth dilution method is the possibility to test a high number of strains in one experiment.

Nisin was the only AMP that completely reduced the growth of three strains at concentrations of 8 µg/ml or less. Thus, it was decided to test higher concentrations of nisin up to 128 µg/ml for other organisms. Consequently, nisin completely inhibited or reduced the growth of 17 strains at concentrations between 1 µg/ml and 128 µg/ml. Ten of these inhibited bacteria are Gram-positive organisms. Nine of the nisin susceptible bacteria are members of the microbial complexes described by Socransky and Haffajee [24] demonstrating that nisin exhibits an antimicrobial activity against the early colonizers, the aggregating bacteria as well as the late colonizers of the dental biofilm. At low concentrations of 8 µg/ml also melittin and lactoferrin reduced some periodontal, Gram-positive and Gram-negative bacteria.

*S. aureus* seemed to be more susceptible to nisin in aerobic than anaerobic conditions while growth on aerobic and anaerobic control plates was identical. This can possibly be explained by an oxygen-dependent gene expression. *S. aureus* shows an increased expression of virulence genes under oxygen depletion conditions [25]. These virulence factors, for example serine-like proteases (splC, splD), can enhance the viability of *S. aureus* in the presence of AMPs [26].

The susceptibility of bacteria to different AMPs depends on the extent of developed resistance as well as the kind and variety of mechanisms of the particular peptide. Nisin’s activity is based on different actions, like pore formation, inhibition of peptidoglycan synthesis and destabilization of the cell wall by oligomerization [27,28]. This functional variety potentiates its antimicrobial activity. LL-37 also seems to interfere in cell wall synthesis beside pore formation and lactoferrin exhibits antimicrobial activity by binding different targets of the outer membrane of bacteria in addition to its iron-binding property [21,29–31]. Nevertheless, for bacteria it seems to be harder to resist nisin’s way of action. *P. micra, Megasphaera* sp. and *B. longum* were the most susceptible bacteria tested against AMPs.

*B. longum*, a member of the intestinal microflora, is inhibited by nisin and melittin. This species is currently of high interest as a probiotic strain [32,33]. The oral administration of *B. longum* and lactobacilli positively modifies the composition of the intestinal microbiota and reduces the inflammatory response in patients with colorectal cancer [34,35]. In addition, *B. longum* could be used as a psychobiotic since it attenuates the physiological and psychological response to acute and long-term stress situations with a subtle improvement in visuospatial memory [36]. Furthermore Yousuf et al. (2017) demonstrated that a daily used probiotic mouth rinse containing *B. longum* and other strains, reduced plaque accumulation and gingival inflammation in schoolchildren [37]. It is of importance that our study demonstrated that this beneficial bacterium is inhibited by the antimicrobial peptide nisin, which is produced by *L. lactis*. Consequently, the concurrent use of both species in one probiotic seems to be questionable. Interestingly, lactoferrin as a natural occurring AMP in the environment of *B. longum* did not reduce its growth. Actually, a dose-dependent growth promotion effect of lactoferrin to some strains of *B. longum* was shown [38]. In other experiments there was no effect on the growth or growth inhibition occurred at concentrations higher than in this study [39,40]. Rahman et al. (2009) suggested that the influence of lactoferrin on the growth could depend on the iron requirement of *B. longum* [38].

In summary, the determined MIC values for all studied AMPs are higher than described in the literature. There is one exception: In 2015 the activity of nisin was tested using the broth microdilution method [41]. Here, the MIC of nisin against *A. odontolyticus* was 10 µg/ml, which is close to our MIC of 16 µg/ml. However, discrepancies persist between the MIC values for *A. actinomycetemcomitans, P. intermedia, S. mutans, F. nucleatum* ssp. *nucleatum* as well as *P. gingivalis* [41]. Other studies analyzed the effect of nisin on *S. aureus* and found either inhibition at MIC values between 2 µg/ml and 32 µg/ml [42] or no growth inhibition [43]. Also melittin showed in different studies antimicrobial activity against *S. aureus* as well as against *E. faecalis* and *E. coli* at relatively low MIC values, mostly under 8 µg/ml [42,44,45]. In our study melittin had no influence on the growth of these bacteria, but reduced the growth of some periodontal strains. Two studies on the activity of parasin-1 found that MIC values ranged between 1 µg/ml and 4 µg/ml for *C. albicans, E. coli, S. aureus* and *S. mutans* [16,46]. Studies of LL-37 detected antimicrobial activity against *A. naeslundii, A. actinomycetemcomitans, E. corrodens, E. faecalis, E. coli, F. nucleatum, P. intermedia, P. aeruginosa* as well as *S. aureus* with MIC values between 0.1 µg/ml and 37.8 µg/ml [47,48]. However, there are studies with much higher MIC values, for example Altmann et al. (2006) determined the MIC of 100 µg/ml for *F. nucleatum* and *A. actinomycetemcomitans* was resistant (MIC >200 µg/ml) in their experiment [49]. In our study no antimicrobial activity could be detected for parasin-1 or LL-37.

Generally, discrepancies of MIC values in the literature could be explained by heterogeneous test conditions. Most of the studies mentioned above used the broth microdilution method according the recommendations of the Clinical and Laboratory Standards Institute (CLSI). Only slight differences occurred concerning the incubation time (overnight, 16 to 48 hours) and the way of reading the results (visible growth, optical density). In our study, an incubation time of 4 days was chosen because many of the periodontopathogenic bacteria are slow growing. The extended incubation time might have compromised the activity of the AMPs. An over time diminishing activity might lead to trailing endpoints [23]. In our experiments no trailing endpoints occurred. However, the most variable factor in all studies is the type of media used for cultivation and for the susceptibility assay. Different compositions and concentrations of additives can influence the susceptibility of bacteria as well as the activity of the AMPs [50]. For instance, bacteria were more susceptible to permeabilization by AMPs when they were grown in a carbonate-containing media before testing [51]. On the other hand the addition of anions to the test media can influence the peptide structure, which in turn correlates with the antimicrobial activity [52,53]. Certainly, it is difficult to compare studies which used a different methodology like the broth dilution, the radial diffusion or the agar dilution method [20]. To our knowledge there are no other studies that used the agar dilution method, although this is the gold standard of anaerobic susceptibility testing [23].

The concentration of lactoferrin in saliva and crevicular fluid strongly varies between 1.5 and 350 µg/ml, whereby higher lactoferrin levels were determined in patients with gingivitis or periodontitis compared to healthy humans [54–57]. In the low range of naturally occurring concentrations lactoferrin already showed an antimicrobial activity against some periodontal bacteria in this study. In contrast, the salivary concentration of LL-37 ranges between 0.22 and 275.00 ng/ml which is much lower than the usually tested MIC values [58,59]. Thus, other biological actions beside the antimicrobial activities could play an additional important role of AMPs in host defense and inflammatory regulation [60]. For example, LL-37 inhibited LPS-induced inflammation and is proposed as a protector against periodontal bone resorption [12,61]. Also lactoferrin showed anti-inflammatory effects; it downregulated LPS-induced bone destruction and inhibited the adhesion of periodontopathogenic bacteria to human cells [62–64].

In addition, an antimicrobial activity against a single bacterial species determined in *in-vitro* experiments does not consequently result in an antibiofilm effect on the complex periodontal multi-species community. However, antibiofilm activities of some AMPs are already detected. Nisin exerted antibiofilm properties against a multi-species preformed biofilm at concentrations of 50 µg/ml which correspond to our determined MIC-values of the middle range [41]. LL-37 inhibited the biofilm formation of *A. actinomycetemcomitans* at concentrations of 20 µg/ml although the bacterium is resistant against the microcidal actions of LL-37 [65]. Consequently, for the antibiofilm activity of AMPs other mechanisms may play a role than the direct bactericidal actions and so even sub-inhibitory concentrations can reduce biofilm formation. For the estimation of the clinical potential as adjuvants in periodontal treatment further multi-species biofilm analysis should be considered.

Critical parameters for the stability of the AMPs are temperature and pH. For this study, all bacteria were cultivated at pH 7 and at 37°C. During the preparation of the plates the AMPs were suspended in liquid agar at a temperature of about 50°C. Obviously nisin could resist such high temperatures showing the highest antimicrobial activity in our study. It has been demonstrated that nisin can survive short-term heat treatments without complete degradation of the peptide structure, even at high temperatures over 100°C [66]. Also melittin and lactoferrin can resist higher temperatures. Ebbensgaard et al. (2015) proved that melittin kept its antimicrobial activity after storing at 90°C for 30 minutes [44]. Suzuki et al. (2003) examined the biological characteristics and the stability of recombinant and native human lactoferrin and showed that the antimicrobial activity of both molecules could be maintained after heat treatments of 65°C [67]. However, the secondary structure of LL-37 is temperature-dependent [68]. The helical conformation of LL-37 seriously defines its mode of interaction with biological membranes and thus also affects the antimicrobial activity [20]. Wildman et al. (2003) showed that the peptide surface orientation at the beginning of the interaction with lipid bilayers is maintained up to 50°C [69]. Studies on the heat stability or temperature-dependent structure of parasin-1 were not found. In summary, a decreased stability of the peptides LL-37 and parasin-1 at higher temperatures cannot be fully excluded and temperature-dependent changes in the structure during the preparation of the agar plates could explain why no antimicrobial activity was detectable.

The ability to tolerate pH variability could be a crucial factor for the therapeutic use of AMPs. In the periodontal pocket the pH can vary in a wide range between 2 and 9 [70]. Nisin and lactoferrin endure low and neutral pH-values; both peptides have a higher thermal stability at low pH-values [66,71,72]. Whereas the activity of nisin decreases with increasing pH, lactoferrin seems to retain its antimicrobial activity over a range of pH between 2 and 7.4 [67,73]. In contrast, the helical structure of LL-37, important for its antimicrobial activity, decreases from pH 5 and is totally degraded at pH 2 [52]. Along with these structural changes, the antimicrobial activity declines at low pH-values [52]. The interaction of melittin with membranes is versatile and very complex; influencing factors are protein-to-lipid ratio, peptide concentration, ionic strength and pH [74–76]. Studies on the antimicrobial activity of melittin and also for parasin-1 in relation to the pH are missing.

## Conclusion

AMPs, especially nisin and to a smaller degree lactoferrin, might be promising alternatives to antibiotics because of their antimicrobial activity, high resistance to environmental conditions and partially low costs.
